# Ankle-Brachial Index and Arterial Stiffness, Modulate the Exertional Capacity of High-Frequency Training Athletes

**DOI:** 10.3390/jcdd9090312

**Published:** 2022-09-19

**Authors:** Raffaello Pellegrino, Eleonora Sparvieri, Andrea Di Blasio, Giovanni Barassi, Massimiliano Murgia, Patrizio Ripari, Angelo Di Iorio

**Affiliations:** 1 Department of Scientific Research, Campus Ludes, Off-Campus Semmelweis University, 6912 Lugano–Pazzallo, Switzerland; 2 Department of Internal Medicine, ASL Teramo, 64100 Teramo, Italy; 3 Department of Medicine and Science of Aging, “G. d’Annunzio” University of Chieti-Pescara, 66100 Chieti, Italy; 4 Center for Physiotherapy, Rehabilitation and Re-education-CeFiRR- Gemelli Molise, 86100 Campobasso, Italy; 5 Department of Anatomical and Histological Sciences, Legal Medicine and Orthopedics, Sapienza University of Rome, 00183 Rome, Italy; 6 Department of Innovative Technologies in Medicine & Dentistry, University “G. d’Annunzio”, 66100 Chieti-Pescara, Italy

**Keywords:** exercise, physical fitness, ankle-brachial index, arterial stiffness, soccer

## Abstract

Ankle-brachial index and arterial stiffness are associated with leg function in the elderly and in patients with peripheral arterial disease. Little is known about the meaning of these parameters in young and trained subjects and how they are related to physical performance. The main objective was to evaluate the mediating role of arterial stiffness and ankle-brachial index in physical performance. In a cross-sectional, case-control study, 240 male athletes were consecutively enrolled from the Laboratory of Cardiology and Sports Medicine, “G. d’Annunzio” University (Italy). All the subjects underwent the examination protocol for the annual medical evaluation for sport participation. Soccer (football) players compared to runners showed a lower level of ankle-brachial index, higher arterial stiffness, and lower systolic and diastolic blood pressure. In the treadmill stress test, soccer players compared to runners showed a greater maximal aerobic capacity. Differences in cardiovascular performance between soccer players and runners were mediated by better arterial stiffness and low level of ankle-brachial index; the estimated effect was 0.11 ± 0.05 and 0.24 ± 0.06, respectively. Vigorous strength training drops blood pressure and increases arterial stiffness. Taken together, our findings would seem to suggest that ABI and CAVI could be used as markers for athletes’ performance.

## 1. Introduction

There is scarce evidence on the physiological and/or performance differences that could help to identify top-level athletes, therefore, the determinants that predict and contribute to high-level performance in sports remain largely unknown [1]. Several markers have been suggested, for example, endurance-related indicators such as the capacity to produce high power outputs even in the presence of fatigue [2], maximal oxygen consumption, lactate threshold [3], and anthropometric indicators [4]. Anthropometric indicators or body mass index are pivotal determinants of the performance of athletes. As a matter of fact, most sports require a specific range in body size and shape. Still, it should be noted, however, that anthropometric measures are commonly reported for groups of specific sports disciplines and have lower capacity to predict performance [4]. Exertional capacity is strictly related to maximal workload achieved, to maximal oxygen consumption, and may be influenced by large artery stiffness [5,6]. Increased aortic stiffness is associated with structural alterations in the vascular media and related to an error in the regulation of endothelium or to an alteration in the vascular smooth muscle tone, and to other aspects of vascular wall structure and function [7]. To balance the homeostatic equilibrium between the metabolic request and the flow supply of oxygen, blood flow and blood pressure increase during a physical strain, exploiting the elasto-mechanical properties of the arteries [8]. To date, is not clear if those cardiovascular indicators can accurately differentiate athletes’ performance. Vigorous training plays an important role in maintaining functional homeostasis [9] and in the long-term adaptation of organs’ apparatus, in their functional reserve, and in physical fitness [10]. There is strong evidence that aerobic exercise is associated with lower arterial stiffness [11], but less is known and the data are controversial about the effects of mixed aerobic–anaerobic training on arterial stiffness and blood pressure variability [12,13]. Moreover, data that analyze the role of arterial stiffness and blood pressure in the level of exertional capacity in trained and active subjects are limited.

**Figure 1:**
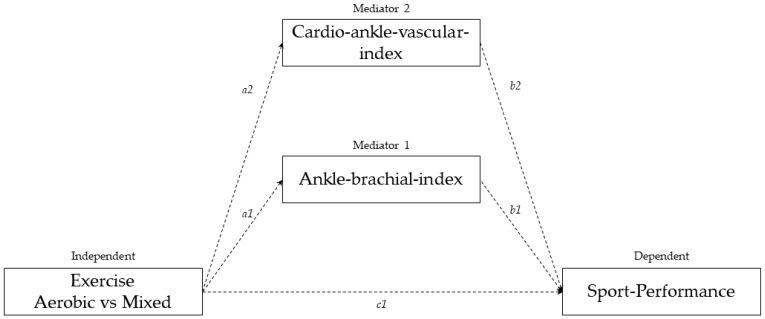
Theoretical model illustrating the hypothesized relationship between the type of training (independent) and sports performance (dependent) according to the mediating role of the ankle-brachial index (M1), and cardio-ankle-vascular index (M2)**.** The c1 notation indicates the association between independent variable and the outcome variables; a1 and a2, show the association between the independent variable and the proposed mediators; b1 and b2 indicate the association direction between dependent and potential mediators.

Our hypothesis considers arterial stiffness and blood pressure as potential cornerstones of the pathway that links the type of exercise training (aerobic vs. mixed aerobic-anaerobic) to physical fitness (Figure 1). Therefore, the main objective of this study is to construct a mediation model to understand if and how arterial stiffness and the ankle-brachial index act on top-training-level athletes’ sport performance, according to type of exercise: aerobic (runners) compared to mixed aerobic–anaerobic (soccer-players).

## 2. Materials and Methods

From the Centre of Sports Medicine of the “G. d’Annunzio” University (Chieti-Pescara, Italy), 240 male top-level athletes (mean age 34.4 ± 14.2) were prospectively selected and recruited, who underwent the annually compulsory medical examination for sport participation and for planning a dietetic and physical training program. Among the study population enrolled, 111 (46.3%) were soccer players (Serie A and B, Italian championship) and 37 (15.4%) were futsal players (Serie A Italian championship). Both groups, underwent a prevalently mixed aerobic–anaerobic training at least 4 times per week (i.e., 12–18 h per week) and were, therefore, collapsed into a single group for further analysis. The remaining 92 subjects were runners, who had a specific aerobic training program, at least 4 times per week, and in the year before, participated in at least at two competitive marathons.

### 2.1. Protocol

The study was designed by the laboratory of Medicine and Sports Cardiology of the University Centre of Sports Medicine of the “G. d’Annunzio” University (Chieti-Pescara, Italy). According to Italian law, all the agonist athletes that are referred to the center for their annual medical visit undergo a clinical examination, a physical performance assessment, and an interview on dietary and lifestyle habits. The research protocol was not submitted to the local ethics review board, since this is the standard assessment in the routinely practice of the center, according to Italian law; nevertheless, written informed consent was obtained from all participants enrolled in the study.

### 2.2. Cardio-Ankle-Vascular Index (CAVI)

CAVI was measured using Vasera VS-1000 (Fukuda Denshi, Tokyo, Japan). Cuffs were applied to the 4 extremities, and electrocardiographic electrodes were attached to the upper arms. A microphone was placed on the sternal angle for phonocardiography. Two amorphous pulse wave sensors were placed symmetrically at the femoral and carotid artery. The subjects rested in the supine position for 5 min. To minimize cuff inflation-effects on blood flow dynamics, pulse waves were measured with cuffs inflated to lower than the diastolic blood pressure (50 mmHg). Then, the extremity blood pressure (BP) was measured by oscillometry. Systolic blood pressure (SBP), diastolic blood pressure (DBP) and pulse pressure (PP) were obtained by measuring the BP at the right brachial artery [14,15]. Vasera also produce lower the limbs’ ankle-brachial index (ABI). The measurement of the ABI involves recording the SBP in the brachial artery at each elbow and SBP in the posterior tibial and the dorsalis pedis arteries at each ankle. The result is reported as a ratio of the ankle systolic pressure in the numerator, over the lower brachial pressure in the denominator. The ABI is calculated for both sides separately, and classified according to standard criteria: (1) normal range (1.0–1.2); (2) acceptable (0.9–1.0); and (3) arterial disease (less than 0.9) [16].

### 2.3. Anthropometry and Body Composition

Body weight and height were measured, to the nearest 0.1 kg and 0.1 cm, respectively, in a standardized position, using a stadiometer with a balance-beam scale (Seca 220, Seca, Hamburg, Germany); subjects were dressed in light clothing and without shoes. Body mass index (BMI) was calculated as weight (kg)/height (m2). The body composition (fat-mass, fat-free-mass, muscle-mass, and total-body-water expressed in Kg) was assessed by means of the electrical bioimpedance technique, using leg-to-leg 50 kHz frequency bioelectrical impedance scale (BC-420MA, Tanita, Tokyo, Japan). The test was executed in a standing position, without socks, after emptying the bladder, and after at least two hours of fasting. Participants also abstained from alcohol consumption within 24 h of the test.

### 2.4. Exercise Treadmill-Stress-Testing

An exercise treadmill test was performed during the morning hours, preceded by a one-minute warm-up at a speed of 1.0 mph, with no slope, and adapted for a tilt treadmill with gradual and moderate increases of speed and slope. The patients were continuously monitored with a 12-lead ECG. The Treadmill exercise test was conducted according to a modified for athletes Bruce protocol, using standard criteria for stopping. The exercise capacity, resting, maximum, and recovery measures of heart rate (HR) were derived from the treadmill test results. Exercise duration was used as an estimate of physical fitness; HR and BP were recorded during each stage of exercise and for each minute of recovery. The rate-pressure product (RPP) was obtained by multiplying the maximum HR by the SBP at peak exertion.

### 2.5. Statistical Analysis

Continuous variables were reported as mean ± standard deviation, and differences between types of sports were analyzed with general linear models. Categorical variables were reported as frequencies and percentages, and the Chi-square test was applied to evaluate statistically significant differences between the groups considered; moreover, logistic regression was applied to produce estimates and to calculate odds ratio (O.R.) and 95% confidence interval (95%CI). Analyses were conducted using growth curve models (linear mixed models) to examine trajectories of BP, HR, and RPP, across time spent on the treadmill test. This analytical procedure address within-person and between-person variability simultaneously. In the Supplemental Web Material, Model A describes how cardiac functional reserve changes linearly over time for each person and how these changes differ across people. The model B and C describe the same changes modeled on a quadratic and cubic function of time spent on the treadmill test, respectively. These three models partitioned and quantified cardiac-functional-reserve variation across both participants and time. The slope for linear, quadratic, and cubic function of time spent on the treadmill test defined as random effects reflected changes in cardiac-functional-reserve, according to each unit increase in time spent on the treadmill test, and also provided the rate of change of the same cardiac- functional-reserve. Deviance statistics, AIC and BIC were also assessed. The second step was to condition the previous higher order term model (i.e., cubic function) with the type of sport practiced and with the two levels of ABI in two different conditional models; the interactions between these variables and the linear, quadratic, and cubic function of the time spent on the treadmill were tested and reported. To test the hypothesis that ABI and CAVI could mediate the effect of the type of sport practiced on time spent in the treadmill stress test were applied a mediation analysis. The procedure requires that four steps must be satisfied to determine mediation [17]. First (c1 in Table 2), a linear regression analysis was used to demonstrate an association between the type of sport and the outcome variables (time spent on the treadmill stress-test). Second, linear regression models were applied to show the association between the type of sport and the proposed mediators (ABI and CAVI: a1 and a2, respectively, in Table 2). Third, a regression model was applied to assess the association between the proposed mediators (ABI and CAVI) and the outcome variable. Lastly, time spent on the treadmill stress test was simultaneously regressed on ABI (b1), CAVI (b2), and the type of sport practiced to determine if the effect of the independent factors on the dependent one was reduced after adjusting for mediators. For the second mediation procedure, the Sobel-test was used, which provides a conservative evaluation of the indirect effect of the independent variable on the dependent variable via the mediator [18]. All analyses were performed using the SAS-software package release 9.4 (SAS Institute, Inc., Cary, NC, USA).

## 3. Results

In the study, we enrolled 240 athletes, 148 (61.7%) and soccer players, all athletes were male. Soccer players compared to runners, showed statistically significant lower levels of CAVI (*p* = 0.01 on the right and *p* = 0.009 on the left side), lower DBP measured at the ankle at the brachial, lower SBP at the ankle, and lastly a lower ABI, on both sides (Table 1).

Between the two groups, no differences could be demonstrated in the body composition and in rest rate metabolism. Compared to runners, soccer players showed more frequently, in both legs, an ABI < 0.9 (OR: 7.0; 95%CI: 2.91–16.92) or an ABI ranging from <1 to >0.9 (OR: 6.2; 95CI: 3.03–12.66). In a sub-analysis considering only soccer players and clustering them according to classic soocer (11 players for every team) compared to Futsal (5 players for every team), the ABI was in the normality range (>1.0) in both legs only in 7/37 (18.9%) for Futsal players and in 48/111 (43.2%) for classic soccer (*p* =0.01).

### 3.1. Type of Sport and Result of the Treadmill Stress Test

**Figure 2:**
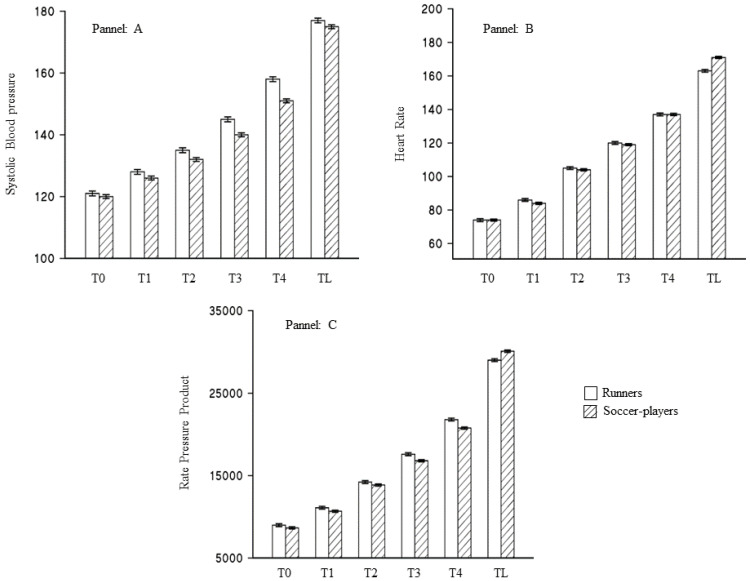
Variation of cardiovascular markers during the treadmill stress test according to the type of sports. Blank boxes represent runners, whereas dashed were soccer players. T0 was the warm-up; TL was the last step reached during the test. In the panel: (**A**) systolic blood pressure (mm/Hg); (**B**) heart rate (bp/min); (**C**) rate pressure product (bp/min*mm/Hg).

SBP, HR, and the RPP were evaluated at every step during the treadmill test, and are all reported in Figure 2, according to type of sport. As could be expected on average, not considering in the models the differences due to the type of sport, SBP, HR, and the RPP showed a departure from linearity. The cubic function was the best choice to represent this relationship as demonstrated by the statistically significant reduction of the goodness-of-fit and by the statistically significant value of the cubic regressors (Supplementary Table S1). When in the three models analyzing the variation of BP, HR, and the RPP, was considered by the type of sport and adjusted for the time needed to reach the maximal aerobic capacity, soccer players showed a statistically significant greater functional performance. Considering the mean value during the test, soccer players compared to runners, showed lower SBP (−6.74 ± 1.83), HR (−0.30 ± 2.53), and RPP (−1.15 ± 0.43); moreover, soccer players, compared to runners, increased their cardiovascular performance more, as demonstrated by the statistically significant interaction between the type of sport and the cubic function of the time spent in the stress test (Supplementary Table S1).

### 3.2. Ankle-Brachial Index and Result of the Treadmill Stress Test

**Figure 3:**
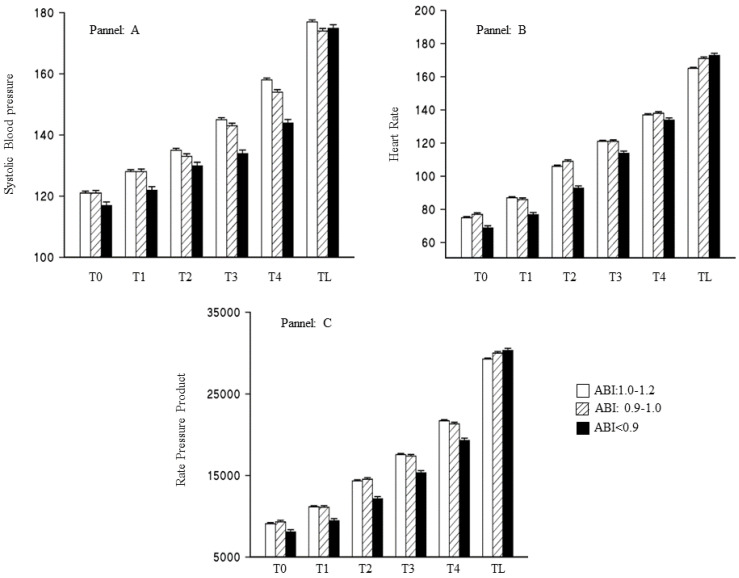
Variation of cardiovascular markers during the treadmill stress test according to the ankle-brachial index. Blank boxes represent subjects with normal values (ABI 1.0–1.2), dashed subjects with acceptable values (ABI 0.9–1.0), and lastly, black box arterial disease (ABI < 0.9). T0 was the warm-up; TL was the last step reached during the test. (**A**) Systolic blood pressure (mm/Hg); (**B**) heart rate (bp/min); (**C**) rate pressure product (bp/min*mm/Hg).

The three markers of cardiac function were also analyzed according to ABI classification (Figure 3). During the stress test, those subjects with an ABI < 0.9 in both legs compared to all other athletes, showed lower SBP (−17.86 ± 2.17; *p* < 0.001), and RPP (−1.82 ± 0.55; *p* < 0.001). Moreover, the trajectory of the SBP and the RPP showed a less pronounced increase in those athletes with an ABI < 0.9 (−8.10 ± 0.86; *p* < 0.001; −0.91 ± 0.24; *p* < 0.001, respectively) even if they showed a statistically significant higher point when the maximal aerobic capacity was reached (Supplementary Table S2).

Heart rate did not show any significant differences between the two groups considered in the stress test (3.71 ± 3.17; *p* = 0.24) and in the threshold at the maximal aerobic capacity (−0.17 ± 0.13; *p* = 0.21); but athletes with an ABI > 0.9, compared to all others, showed a more pronounced increase in heart rate (3.28 ± 1.37; *p* = 0.02) at every step (Supplementary Table S2). When the same analysis was repeated for CAVI, no statistically significant interaction term between the arterial stiffness and time could be demonstrated for BP and for RPP. No statistically significant differences could be shown for the average value of HR during the test (−3.17 ± 1.90; *p* = 0.09); but at every step for a lower level of CAVI, the increase in HR was less pronounced (5.41 ± 2.38; *p* = 0.02), and the threshold at the maximal aerobic capacity was higher (0.17 ± 0.08; *p* = 0.03).

### 3.3. Mediation Analysis

In Table 2, we report the analysis used to evaluate the role of CAVI and ABI in the mediation of the association between time spent in the treadmill stress test and type of sport practiced. Both the potential mediators were significantly predicted by the type of sport (a1, a2 in Table 2); ABI (b1) and CAVI (b2) predicted, independently by type of sport, the time spent in the treadmill stress test. Lastly, soccer players spent a longer time on the treadmill stress test (c1). The total combined direct effect was estimated to be 0.35 ± 0.07 (95%CI: 0.23–0.50), whereas the effect to be attributable to ABI was 0.24 ± 0.06 (95%CI: 0.15–0.37) and the effect due to CAVI was 0.11 ± 0.05 (95%CI: 0.03–0.21). The Sobel test for the evaluation of the mediation effect was statistically significant (*p* < 0.001).

## 4. Discussion

In this study of a well trained and active young population, we found that lower ankle-brachial index, and lower level of Cardio-ankle-vascular index, could mediate differences in physical performance between soccer players and runners; moreover, in those subjects who play football, it could be demonstrated a more prevalent lower level of the ankle-brachial index compared to runners.

In our theoretical model, ABI and CAVI were considered as mediators of physical performance, therefore rather than hypothesizing a direct causal relationship between the independent (type of exercise) and the dependent variable (physical performance), the role of a third variable, the mediator, was analyzed to clarify the nature of this relationship. Endothelial function, measured by ABI and CAVI, together explains 35% of the difference in physical performance between “pure” aerobic training (runners) compared to concurrent training (soccer players). Numerous cross-sectional and longitudinal intervention studies have demonstrated the influence of regular aerobic training on vascular function in different populations [19,20]. Controversial reports have investigated the relationship between arterial stiffness and strength-based training; some authors have found a more pronounced arterial stiffness in strength-trained men compared to sedentary volunteers [21]; on the contrary, no differences in pulse wave velocity were reported between similar study groups by others [22]. Moreover, a confounding effect in the variation of arterial stiffness during strength-training program was attributed only to age, gender and race [23]. Runners enrolled in the study did not show statistically significant differences in body composition and in heart rate at rest compared to soccer players, as could be expected due to the type and intensity of training that they perform [24], therefore a lower level of fitness could be hypothesized for the former group of athletes. Taking into account this hypothesis, ABI and CAVI could be considered as markers of cardiovascular performance.

Ankle-brachial index, particularly in the elderly, was largely recognized as a good predictor of physical function. In a case-control study, McDermott demonstrated that ABI has a direct and independent association with walking endurance and both usual and maximal walking velocity [25]. In the context of this study, analyzing a group of young and trained athletes could be identified an inverse association between ABI and performance, supporting the notion that athletes with low levels of ABI have a better cardiovascular performance. An ABI lower then 0.9 was reported in the literature as a marker of peripheral arterial disease, but none of the subjects enrolled in this study reported symptoms such as claudicatio, leg pain that does not go away when the exercise was stopped, foot or toe wounds, and a marked decrease in the temperature of the lower limb, particularly compared to the other leg or to the rest of the body.

A reason that can explain this inverse relationship could be related to the type of exercise that soccer players did, and the consequent modification on the type of metabolism, on body composition, and lastly on cardiovascular system adaptations. During exercise training, soccer players alternate high-speed runs, sprints, turns, jumps, and tackles, which provide a high impact on muscles and bones. From a metabolic point of view, this kind of exercise enhances fat oxidation during activity and results in a fat mass reduction [26] with a positive effect on muscle and fat mass balance. An alternative explanation could also be found in the nitric oxide (NO) metabolism; there is concrete evidence that physical activity enhances NO production [27], and NO metabolic pathway positively affects indices of cardiovascular health and exercise tolerance. It has also been reported that NO could lower blood pressure in hypertensive patients, and can improve vascular endothelial function, and reduce the stiffening of large elastic arteries [28].

In our data, we could not find statistically significant differences between endurance and concurrent training in body composition and basal metabolism, therefore variation in rate and type of metabolism could not be demonstrated and consequently could not be considered as a keystroke in our model, even if the beneficial effect of soccer training was largely proved in young as in the elderly and in both sex [29].

The exercise type done by soccer players could determine a reduction of BP, and in our data, particularly in the lower limb. Several mechanism could be considered in this process in response to physical training in soccer players: modulation in the autonomic nervous system, reductions in systemic vascular resistance, and plasma hormonal level [26,30]. In a clinical trial designed to analyze the effect of soccer training, in an elderly ever untrained population, was demonstrated an increase in muscle capillarization (23%), moreover the same researchers in a similar study group demonstrated also a significant reduction in the augmentation index, a marker of arterial stiffness [29]. All together this data supports the notion that soccer players could have a lower blood-pressure at the ankle, probably through an increase of the capillarization and/or through a reduction of the arterial stiffness. Nevertheless, other vascular structural adaptations may have occurred, i.e., increased length, cross-sectional area and/or diameter of already existing arteries and veins. The hypothesis that the type and intensity of exercise could mediate physical performance through vascular remodeling was moreover supported by the fact that futsal players showed a higher percentage of low ABI level (35%), compared to soccer players (21%) and runners (8%). Futsal requires a more intense activity compared to soccer; sprints, change of direction, jumps, and tackles are more intense and frequent, moreover inducing a significant higher loading of the aerobic system (HR response), and lastly it is more difficult to perform technical actions. Altogether, this data supports the hypothesis that a dose/response model could be hypothesized among the type and intensity of exercise training and blood pressure reduction.

Some limitations of this study must be acknowledged. A selection bias could be hypothesized; indeed, the soccer players were almost all pro or semi-pro, whereas among runners, almost all were amateurs, therefore, the former group could be less trained, or alternatively, be trained without a specific help of a qualified coach. Moreover, no standardized information was available about the intensity and regularity of the runners’ training. In any case, in this study, we enrolled only runners that belonged to a sportive association, and in the year before the enrolment in the study, had participated in at least two competitive marathons, ensuring regular and constant training.

Another limitation that must be considered was the lack of a direct measurement of cardiac oxygen consumption. In all the analyses, we used the rate-pressure-product, that was largely used in the literature and reported to correlate quite well with maximal oxygen consumption in a wide variety of circumstances [31].

In those subjects with an ABI level < 0.9, it was not possible to have a computed tomographic scan or magnetic resonance angiography because none of the athletes reported a paradigmatic symptom of peripheral-arterial-disease, nor was it possible to identify any significant reduction in blood flow at the Doppler and ultrasound images. Lastly, we did not consider in our analysis several well-known cardiovascular risk factors such as smoking habits, cholesterol, or vitamin D levels, as those data were not available for all the enrolled subjects. However, arterial stiffness is now regarded as an important biomarker of vascular function and a predictor of cardiovascular events more accurate than well-established traditional risk factor scores [32].

## 5. Conclusions

Concurrent aerobic–anaerobic exercise compared to pure aerobic training appear to improve endothelial function; moreover, arterial stiffness (CAVI) and ankle-brachial index exert a positive effect on performance in the treadmill stress test. Lastly, CAVI and ABI could be suggested as determinants that could predict and contribute to high-level performance in sports.

## Figures and Tables

**Table 1:** Systematic differences between runners and soccer players in body composition, blood pressure, and in arterial stiffness.

Variables	Runners 92	Soccer-Players 148	*p*-Value
Age (years)	23.52 (3.76)	23.58 (3.91)	0.91
Height (m)	1.77 (0.06)	1.79 (0.06)	0.63
Weight (Kg)	75.66 (6.34)	75.33 (6.95)	0.71
Cardio ankle vascular index right	6.01 (0.59)	5.80 (0.64)	0.01
Cardio ankle vascular index left	5.98 (0.63)	5.75 (0.69)	0.009
SBP brachial right (mm/Hg) ^1^	124.55 (6.86)	124.22 (6.64)	0.71
DBP brachial right (mm/Hg) ^1^	78.76 (9.53)	69.10 (5.96)	0.001
SBP ankle right (mm/Hg) ^1^	129.54 (12.04)	124.12 (13.29)	0.002
DBP ankle right (mm/Hg) ^1^	76.97 (10.74)	69.56 (6.70)	0.001
Ankle-brachial index right	1.04 (0.09)	1.00 (0.10)	0.009
SBP brachial left (mm/Hg) ^1^	125.45 (5.38)	125.14 (5.38)	0.66
DBP brachial left (mm/Hg) ^1^	78.18 (8.44)	69.26 (6.10)	0.001
SBP ankle left (mm/Hg) ^1^	129.51 (8.97)	122.91 (11.35)	0.001
DBP ankle left (mm/Hg) ^1^	77.66 (8.41)	69.16 (6.72)	0.001
Ankle-brachial index left	1.03 (0.07)	0.98 (0.08)	0.001
Rest rate metabolism	1964.89 (163.43)	1988.87 (157.46)	0.26
Fat mass (percentage)	14.18 (3.78)	14.00 (3.37)	0.70
Muscle mass (percentage)	81.49 (3.58)	81.70 (3.20)	0.64

^1^ Tables: systolic blood pressure (SBP); diastolic blood pressure (DBP).

**Table 2:** Path coefficients of the model estimating the mediation effect of the ankle-brachial index (ABI) and arterial stiffness (CAVI) in the association between time spent in the treadmill stress test and type of sport practiced.

	Path	Coefficient	Standard Error	*p*-Value
Type of sport predicting ankle-brachial index(ABI)	a_1_	−0.05	0.01	<0.001
Type of sport predicting arterial stiffness (CAVI)	a_2_	−0.22	0.09	0.01
Ankle-brachial index predicting time spent in the treadmill stress test	b_1_	−11.33	1.50	<0.001
Arterial stiffness (CAVI) predicting time spent in the treadmill stress test	b_2_	−1.14	0.18	0.008
Direct effect of type of sport on time spent in the treadmill stress test	c_1_	1.48	0.24	<0.001

The c1 notation indicates the association between independent variable and the outcome variables; a1 and a2, show the association between the independent variable and the proposed mediators; b1 and b2 indicate the association direction between dependent and potential mediators.

## Data Availability

The datasets used and/or analyzed during the current study are available from the corresponding author upon reasonable request.

## Supplementary Materials

The following supporting information can be downloaded at: https://www.mdpi.com/article/10.3390/jcdd9090312/s1, Table S1: variation of cardiovascular markers during the treadmill-stress-test; model comparisons to test departure from linear decoy. Table S2: Variation in blood pressure, heart rate and in the rate pressure product during the Treadmill Stress Test (TST) according to Ankle brachial index
