# Supplementary material for: Ankle-Brachial Index and Arterial Stiffness, Modulate the Exertional Capacity of High-Frequency Training Athletes
Source: J Cardiovasc Dev Dis. 2022 Sep 19;9(9):312. doi: 10.3390/jcdd9090312 (PMC9506274; doi:10.3390/jcdd9090312)
Supplement: Supplementary file 1 [file jcdd-09-00312-s001.zip › jcdd-1884782-supplementary.pdf]

Supplementary Table S1: Variation in blood pressure, heart rate and rate pressure product during the Treadmill Stress Test according to type of sport.

*

	Blood Pressure	Heart rate	Rate Pressure Product
Fixed			
Intercept	115.32 (1.98)*	61.14 (2.53)*	6.86 (0.46)*
Time (linear term)	8.23 (1.64)*	18.40 (2.54)*	3.58 (0.41)*
Time (quadratic term)	-1.02 (0.58)	-1.72 (0.87)‡	-0.60 (0.15)*
Time (cubic term)	0.24 (0.06)*	0.27 (0.08)†	0.10 (0.01)*
Time spent in the TST	-0.16 (0.13)	-0.43 (0.15)†	-0.10 (0.03)*
Rate of change			
Runners vs soccer	-6.74 (1.83)*	-0.30 (2.53)	-1.15 (0.43)†
Sport * time (linear)	9.48 (2.08)*	2.30 (3.23)	2.04 (0.52)*
Sport * time (quadratic)	-3.95 (0.74)*	-1.58 (1.10)‡	-0.91 (0.18)*
Sport * time (cubic)	0.41 (0.07)*	0.24 (0.10)*	0.11 (0.02)*
Variance Components			
Within Person	13.24 (0.86)	27.38 (1.44)*	0.81 (0.04)*
In 1st grade status	13.47 (20.29)	0.01 (0.01)	0.01 (0.01)
Linear term			
Variance	22.65 (26.70)	129.80 (29.17)*	1.66 (0.69)‡
Covar with 1st grade status	-14.72 (22.64)	-40.30 (11.83)*	-0.61 (0.29)†
Quadratic Term			
Variance	8.20 (3.20)†	21.68 (4.16)*	0.58 (0.10)*
Covar with 1st grade status	13.86 (7.68)‡	22.88 (5.12)*	0.78 (0.13)*
Covar with linear term	-15.43 (9.13)‡	-52.90 (10.86)*	-1.10 (0.25)*
Cubic Term			
Variance	0.11 (0.03)*	0.22 (0.04)*	0.008 (0.001)*
Covar with 1st grade status	-1.89 (0.75)‡	-2.54 (0.56)*	-0.11 (0.02)*
Covar with linear term	1.98 (0.89)‡	5.20 (1.09)*	0.14 (0.03)*
Muscle mass (percentage)	81.49 (3.58)	81.70 (3.20)	0.64

p<0.001; † p<0.01; ‡ p<0.05

Supplementary Table S2: Variation in blood pressure, heart rate and in the rate pressure product during the Treadmill Stress Test (TST) according to Ankle brachial index.

	Blood Pressure	Heart rate	Rate Pressure Product
Fixed			
Intercept	113.22 (1.75)*	56.35 (2.17)*	5.88 (0.39)*
Time (linear term)	10.40 (1.07)*	22.28 (1.69)*	4.56 (0.29)*
Time (quadratic term)	-1.97 (0.40)*	-3.30 (0.59)*	-1.00 (0.10)*
Time (cubic term)	0.33 (0.04)*	0.45 (0.06)*	0.15 (0.01)*
Time spent in the TST	-0.10 (0.14)	4.56 (0.70)*	0.50 (0.13)‡
Runners vs soccer	0.92 (0.61)	-0.33 (0.16)‡	-0.07 (0.03)*
Rate of change			
ABI <0.9 vs ABI≥0.9	-17.86 (2.17)*	3.71 (3.17)	-1.82 (0.55)*
ABI * time (linear)	20.06 (2.49)*	-13.46 (3.94)*	1.52 (0.67)‡
ABI * time (quadratic)	-8.10 (0.86)*	3.28 (1.37)‡	-0.91 (0.24)*
ABI * time (cubic)	0.87 (0.09)*	-0.17 (0.13)	0.12 (0.03)*
**Variance Components**			
Within Person	12.74 (0.67)*	27.45 (1.45)*	0.81 (0.04)*
In 1st grade status	0.01 (0.01)	0.01 (0.01)	0.01 (0.01)
**Linear term**			
Variance	7.93 (9.47)	93.22 (26.64)*	2.29 (0.69)*
Covar with 1st grade status	-0.28 (4.16)	-28.43 (11.17)‡	-0.90 (0.29)‡
**Quadratic Term**			
Variance	4.75 (1.30)*	19.88 (4.04)*	0.64 (0.11)*
Covar with 1st grade status	6.61 (1.70)†	21.11 (5.03)*	0.85 (0.13)*
Covar with linear term	-7.99 (3.38)*	-44.22 (10.26)*	-1.32 (0.27)*
**Cubic Term**			
Variance	0.07 (0.02)*	0.23 (0.04)*	0.008 (0.001)*
Covar with 1st grade status	-1.00 (0.20)‡	-2.63 (0.57)*	-0.11 (0.02)*
Covar with linear term	1.06 (0.34)*	4.79 (1.05)*	0.16 (0.03)*
Covar with quadratic term	-0.57 (0.14)*	-2.13 (0.42)*	-0.07 (0.01)*

* p<0.001; † p<0.01; ‡ p<0.05
